# Estimating human-to-human transmissibility of hepatitis A virus in an outbreak at an elementary school in China, 2011

**DOI:** 10.1371/journal.pone.0204201

**Published:** 2018-09-24

**Authors:** Xu-Sheng Zhang, Giovanni Lo Iacono

**Affiliations:** 1 National Infection Service, Centre for Infectious Disease Surveillance and Control, Public Health England, London, United Kingdom; 2 Medical Research Council Centre for Outbreak Analysis and Modelling, Department of Infectious Disease Epidemiology, Imperial College School of Public Health, Norfolk Place, London, United Kingdom; 3 Chemical and Environmental Effects Centre for Radiation, Chemical and Environmental Hazards, Public Health England, London, United Kingdom; 4 School of Veterinary Medicine, University of Surrey, Guildford, United Kingdom; The University of Hong Kong, CHINA

## Abstract

Hepatitis A is caused by hepatitis A virus and occurs worldwide. Estimating the transmissibility, which is usually characterized by the basic reproductive number *R*_0_, the mean number of secondary infectious cases generated by a single primary infectious case introduced into a totally susceptible population, provides crucial information for the effort required to stop infection spreading. Hepatitis A virus is usually transmitted indirectly through contaminated food and environment. An outbreak from March to June 2011 was reported to have occurred at an elementary school of 698 pupils in China and it was found that the outbreak was due to direct transmission between school children. Based on the symptom onset date and the social contact network of the children, in this study we estimate the serial interval (i.e. the gap in symptom onset between an infectee and its infector) and use different statistical methods to estimate *R*_0_. Combining with the positivity of IgG antibodies tests, we develop a compartmental transmission dynamics model which includes both asymptomatic and symptomatic infections to estimate the overall *R*_0_. Our analysis suggests a serial interval of mean = 23.9 days and standard deviation = 20.9 days. The different statistical methods suggest estimates for *R*_0_ in the outbreak varying from 2.1 to 2.8, and the estimates from the transmission dynamics model are consistent with this range. Our estimates are in agreement with that from one study in England but are higher than that from one study in the United States. Our transmission dynamics model suggests that the proportion of symptomatic infections is about 9%, implying that there were about 344 asymptomatic infections along with the 32 observed symptomatic cases. Furthermore, it is shown that the inclusion of asymptomatic infection in the epidemic process increases the estimate of *R*_0_ but does not do so greatly provided that the proportion of symptomatic infections is constant over the outbreak and there is no difference in transmissibility between symptomatic and asymptomatic infections.

## Introduction

Hepatitis A is a disease caused by hepatitis A virus (HAV). Most outbreaks of hepatitis A have been linked to consumption of uncooked contaminated foods, and contaminated waters. It was noted that some outbreaks are propagated through close contact between humans (e.g., [1,2]). Wu et al [3] reported an outbreak at an elementary school of 698 pupils in Anhui province, China. Twenty-eight serologically confirmed cases aged between 7 and 13 years with symptom onset from 7 May to 8 June 2011 were reported in the school. The social network analysis found that all these 28 cases were connected to a cluster of three pupils with clinical symptom onset on 23, 30 March and 10 April through direct contact or a shared social setting. These three cases were presumed to link to a 4-year-old boy (described as boy A) who had symptom onset on 10 March and was the most probable source case. The investigations provided evidence that this outbreak was due to human-to-human transmission.

Vaccination is the best way to prevent Hepatitis A. All of the cases in this outbreak (except boy A) were children born before 2006 and had not received free vaccination which was introduced in 2006 in Anhui province, China [3]. All the 32 cases had their symptom onset dates recorded and the network diagram presented in Fig 1 of [3] showed that there were 15 cases who had a uniquely known infector. To effectively control the spread and outbreak of hepatitis A, it is important to know its transmissibility which will help estimate the proportion of children that have to be vaccinated to create herd immunity that would protect the whole community. Wu et al [3] also noticed that the only five sporadic cases of HAV infection identified during 2004–2010 in the town at which the school was located were adults aged >25 years. It is well known that many HAV infections in children are asymptomatic [4,5]. Wu et al. [3] reported that about 40% of the symptomatic cases did not recall any close contact with jaundiced persons and environmental sources of infection were not justified. Wu et al [3] further found that 53.5% of 144 pupils at the school who were without symptoms after the outbreak were positive for the IgG antibodies tests. These two facts suggest that transmission occurs from asymptomatic cases as well as from symptomatic cases. This study [3] for the outbreak provides a good dataset to estimate the transmissibility of hepatitis A virus among naïve young children, which is the objective of this study.

In general, the transmissibility of an infectious agent is usually characterised by the basic reproductive number (*R*_0_), the mean number of secondary infections caused by a single primary infectious individual introduced into a completely susceptible population [6]. It is a threshold parameter: if *R*_0_<1, the disease dies out without any intervention; otherwise, the disease can persist and cause epidemics. Another relevant parameter is the effective reproductive number (*R*_t_), the average number of secondary infections caused by a single infective individual introduced into a population made up of both susceptible and non-susceptible individuals. *R*_t_ is a key parameter that describes the state of an epidemic and is important for judging whether the intervention is working to keep the infection under control [7].

In this study we use three statistical methods to estimate *R*_0_ and these are based on the symptomatic infections. A fourth method that we propose is a transmission dynamics model which includes both asymptomatic and symptomatic infections. The consistency between results will provide cross-validation of the different estimation approaches and show the suitability of using symptomatic infection data alone in estimating *R*_0_ [8]. For simplicity, in this study we assume symptom onset coincides with the onset of infectiousness, and so we approximate the generation time (the average duration between the time of infection of each infected case and the time of infection of their infector) by the serial interval (the average difference in symptom onset dates of infector-infectee pairs).

## Methods

We use three different statistical methods (Table 1) to estimate the basic reproductive number *R*_0_ and related quantities. These are i) Transmission tree method; ii) Renewal equation method; and iii) General growth method. For these statistical methods, asymptomatic infections are ignored. We also use transmission dynamics model to describe the asymptomatic and symptomatic infections and to cross-validate the above statistical methods.

**Table 1:** Comparison of different methods for estimating the transmissibility *R*_0_.

Method	Transmission Tree	Renewal equation	General growth	Transmission dynamics
Data required	Incidence data (symptom onset dates); contact information	Incidence data; Serial interval	Incidence data during the early phase of an outbreak; Serial interval	Incidence data; periods of life history stage, positivity of IgG test
output	transmission tree, *R*_*t*_ and serial interval distribution	*R*_0_ and control point *t*_c_	Deceleration parameter (*p*), growth rate and thus *R*_0_ by Eq (7)	*R*_0_, control point *t*_c_, reduction in contact (*ω*), proportion of symptomatic infection (*ρ*)
Estimate of ***R***_**0**_	2.10 [1.90,2.30]	2.82[1.70,4.50]	2.35[2.24,2.48]	2.69 [1.90,3.92]

The direct estimate of serial interval distributions (mean = 23.9days, SD = 20.9days) are assumed. For the method of transmission tree, result was obtained by assuming the time turning point at 26 May 2011, which is obtained from Renewal equation method.

### Data

**Fig 1:**
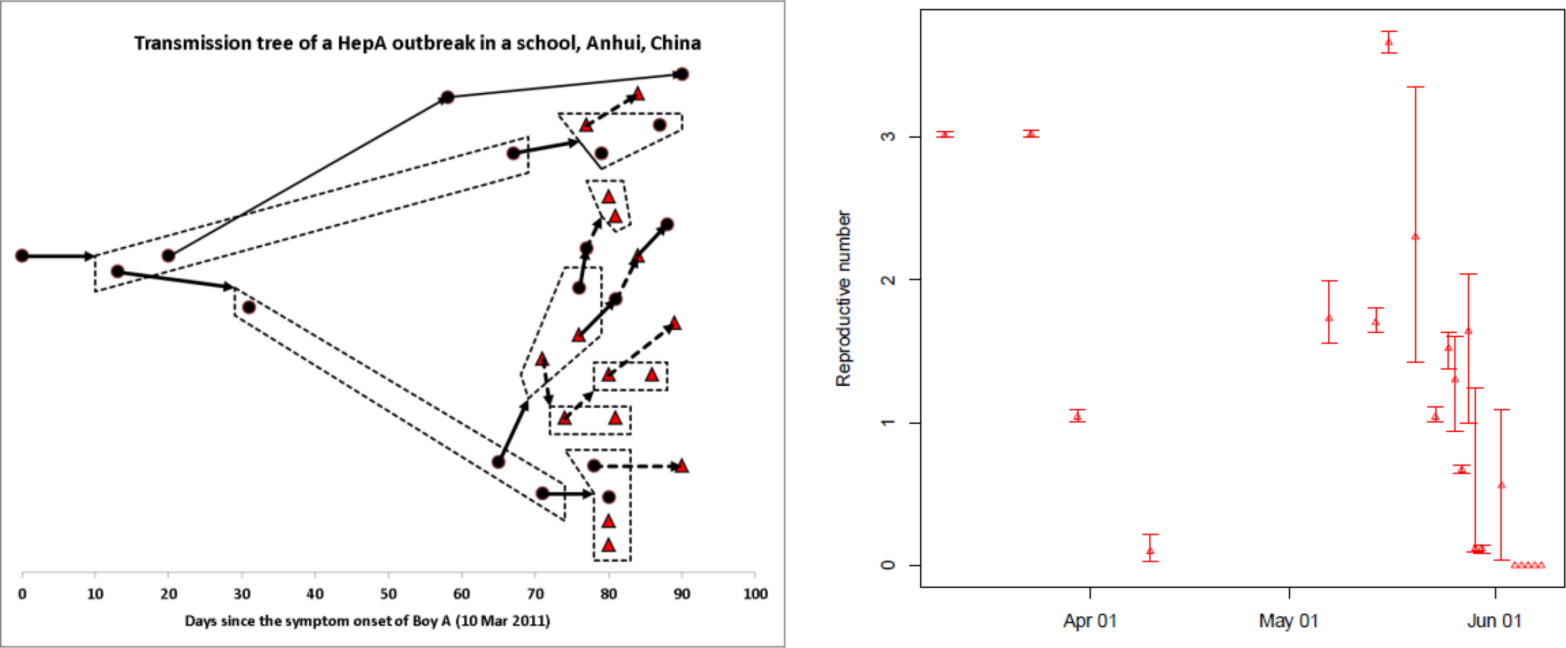
**One sample transmission tree (left panel) and the effective reproductive number along the course of outbreak (right panel).** In right panel, the filled triangles represent mean and bars the lower and upper levels of 95% Confidence interval (CI). In the transmission tree which describes who acquired infection from whom among 32 cases, 15 cases (black circles except index case) know their unique infectors and the infectors of other 16 cases (red triangles) were reconstructed by the method of [9]. The dashed lines were used to enclose infectees of infector (if there is more than one infectee).

The Information about the contact and symptom onset of 32 cases has been extracted from Figs 1 and 2 of [3]. The details are listed in Table 2.

**Table 2:** Outbreak data extracted from [3].

case	Date of onset	Suspected source of infection	Case	Date of onset	Suspected source of infection
1	10 Mar 2011	–	17	29 May 2011	7,8
2	23 Mar 2011	1	18	29 May 2011	10,11,16
3	30 Mar 2011	1	19	29 May 2011	12,14
4	10 Apr 2011	2	20	29 May 2011	8,16
5	7 May 2011	3	21	29 May 2011	1,5,8
6	14 May 2011	2	22	30 May 2011	4,14
7	16 May 2011	1	23	30 May 2011	9,16
8	20 May 2011	2	24	30 May 2011	11
9	20 May 2011	unknown	25	2 Jun 2011	unknown
10	23 May 2011	unknown	26	2 Jun 2011	3,13,18
11	25 May 2011	unknown	27	4 Jun 2011	unknown
12	25 May 2011	6	28	5 Jun 2011	7
13	26 May 2011	7,9	29	6 Jun 2011	25
14	26 May 2011	12	30	7 Jun 2011	13,18
15	27 May 2011	8	31	8 Jun 2011	10,15
16	28 May 2011	7	32	8 Jun 2011	5

### Statistical estimation

#### Transmission tree method

We first re-construct the transmission tree during the outbreak and estimate serial interval, and evolution of transmissibility along the outbreak. The method [9] is a development of method of [10] by further combining partly known contact information among cases with the time intervals in symptom onset between cases to construct the transmission tree. The general idea is to estimate the probability *p*_*ij*_(**v;w;θ**), that case *j* is the infector of case *i*, (where *j =* 1 is the index case) given the duration |*t*_*i*_-*t*_*j*_| between symptom onset of case *i* and case *j*, given the information on the known possible infector **v** (*i*.*e*. a vector representing the case identifiers of the infector of case 2,…,*n*) and the known contacts **w** (*i*.*e*. a vector representing the case identifiers of the contacts of case 2,…,*n*). The method also assumes that observed serial intervals are positive random variables described by a serial interval distribution, g(Δ*t* |**θ**), here assumed to be a gamma distribution with parameter set **θ** = {shape, rate}. The probability *p*_*ij*_, can be calculated as that of observing the duration between the symptom onsets in cases *i* and *j*, g(*t*_i_-*t*_j_|**θ**), times the probability of a potentially infectious contact between *i* and *j*, *π*_*ij*_, normalized by the probability of *i* being infected by any other case *k*:

$$p_{ij}(v,w;\theta )=\frac{\pi_{ij}(v,w)g(t_{i}-t_{j}|\theta )}{\sum_{k\neq i}\pi_{ik}(v,w)g(t_{i}-t_{k}|\theta )}$$ (1)

The probability, *π*_*ij*_, is based on the contact information (**v**,**w**) of both cases *i* and *j* collected during the outbreak (see Table 2). If case *i* has “suspected source(s) of infection”, *π*_*ij*_ = 1/(number of suspected sources of infection) for *j* equal to one of the “suspected source of infection” and *π*_*ij*_ = 0 otherwise. If “suspected source of infection” is “unknown” for case *i*, *π*_*ij*_ = 1/(number of cases with onset before case *i*) for case *j* that has symptom onset before case *i*, and *π*_*ij*_ = 0 for others *j*.

Following [9], the total log-likelihood of a transmission tree of the outbreak size *n* is given by:

$$L(\theta |t,v,w)=\sum_{i=2}^{n}\sum_{j\neq i}^{n}p_{ij}(v,w;\theta )\log[g(t_{i}-t_{j}|\theta )]$$ (2)

The method can simultaneously estimate the most likely transmission tree and the distribution of serial interval (i.e., the shape and rate parameters of gamma distribution). The effective reproductive numbers along the outbreak can then be estimated by summing over all the infectious contacts up to time *t* [10],

$$R_{t}=\sum_{j}\sum_{i=2}p_{ij}(v,w;\hat{\theta })$$ (3)

Where the parameter set $\hat{\theta }$ is inferred by maximizing the total log-likelihood of the data *L*(**θ**|*t*,**v**,**w**) [9].

#### Renewal equation method

In the simplest formulation of the renewal equation [11], the epidemic grows exponentially to infinity. Wu et al [3] reported that some interventions had been taken to control the continuing infection such as isolating affected patients in hospitals, placing members of these cases’ families and other contacts under medical observations, and recommending pupils to take basic hygiene measures. These and other interventions should have been involved during the outbreak and they might start at different times. Here we model the interventions by simplifying the complex processes and assume that they collectively acted to make a change in contact rate and thus transmissibility at some time point *t*_c_. Let *c*_*t*_ be the number of cases whose symptom onset at day *t*, its expected value is approximated by

$$\begin{array}{l} E(c_{t})=R_{0}\sum_{0<t-s}c_{t-s}w_{s},\;t<t_{c};\\ E(c_{t})=R_{0}\sum_{0<t-s<t_{c}}c_{t-s}w_{s}+R_{c}\sum_{t-s\geq t_{c}}c_{t-s}w_{s},\;t\geq t_{c}. \end{array}$$ (4)

Here *w*_*s*_ represent the probability mass function of the serial interval of length *s* days, which can be obtained by

$$w_{s}=G(s)-G(s-1)$$ (5)

with G(.) representing the cumulative distribution function of the gamma distribution. After the turning point *t*_c_, the reproductive number reduces from *R*_0_ to *R*_c_. Here *R*_0_ represents the reproductive number in the absence of any control or susceptible depletion and the value of *R*_c_ shows the impact of countermeasures. Neglecting depletion of susceptibles is justified by the fact that the number of infected individuals is very small compared to the total number of pupils (28 *vs* 698). (It is worth noticing that in statistical estimation methods all infections are assumed to be symptomatic) This allows using of *R*_0_ instead of the instantaneous reproductive number (the average number of people someone infected at time *t* can infect over their entire infectious lifespan) as originally used in [11]. More complicated modifications have been proposed for the renewal equation to reflect the outbreaks that will finish with many individuals still being susceptible (e.g., [12]).

We assume that the variation in daily case counts is captured by Poisson distribution (c.f. [13]). Given the serial interval, the three parameters to be estimated are the reproductive numbers *R*_0_ and *R*_c_ and the turning point *t*_c_. Within the Bayesian framework, Monte-Carlo Markov chain (MCMC) samplings are used to obtain their posterior distributions.

#### General growth rate method

We consider a general-growth model [14] to characterise the ascending phase of the outbreak by assuming the incidence Δ*C*(*t*) at day *t* is given by

$$\Delta C(t)=rC{\left(t\right)}^{p}$$ (6)

where *C*(*t*) describes the cumulative number of cases at day *t*, *r* is a positive parameter denoting the growth rate, and *p*ϵ[0.1] is a ‘deceleration of growth’ parameter (dimensionless). When *p* = 1, it describes exponential growth in the Malthus equation: *C*(*t*) = *C*_0_ exp(*rt*). Here *C*_0_ is the initial number of cases. The basic reproductive number, *R*_0_^exp^ for exponential growth [15], can be approximated by the average number of secondary cases generated by initial cases during the first generation interval *T*_g_ (assumed to be fixed) as,

$$R_{0}^{\exp}=\exp(rT_{g})$$ (7)

For sub-exponential growth models (i.e., 0≤*p*<1), Chowell et al [14] illustrated an equivalent approach to derive the relevant formulae of *R*_0_. As in Renewal equation method, the three parameters *r*, *p* and *C*_0_ will be estimated by Bayesian inference, and the reproductive number can be further obtained.

### Transmission dynamics model

We consider the realistic situation where not all infections are symptomatic. Susceptible pupils (*S*) contacts with infections and then become infected but not yet infectious (*E*). The exposed people progress to become infectious but not symptomatic (*I*) at rate *σ*. This infectious stage is occult. A proportion of occult infection (*ρ*) become symptomatic (*D*) and the rest remain asymptomatic but infectious (*A*). All infections recover and become immune to HAV. Occult infections that do not accompany any readily discernible symptoms progress to become symptomatic at rate *γ*_1_, and the diseased infections recover to become immune at rate *γ*_2_. Both asymptomatic and symptomatic cases are assumed to be indistinguishable in their infectivity and their life history. Therefore the mean of total infectious period is 1/*γ*_1_ + 1/*γ*_2_, and the mean incubation period is 1/*σ* + 1/*γ*_1_. The transmission dynamics model is described by the following differential equations

$$\begin{array}{l} \frac{dS(t)}{dt}=-\omega (t)\beta \frac{I(t)+A(t)+D(t)}{N}S(t)\\ \frac{dE(t)}{dt}=\omega (t)\beta \frac{I(t)+A(t)+D(t)}{N}S(t)-\sigma E(t)\\ \frac{dI(t)}{dt}=\sigma E(t)-\gamma_{1}I(t)\\ \frac{dA(t)}{dt}=\gamma_{1}(1-\rho )I(t)-\gamma_{2}A(t)\\ \frac{dD(t)}{dt}=\gamma_{1}\rho I(t)-\gamma_{2}D(t) \end{array}$$ (8)

In the above equation parameter *ω*(*t*) is introduced to reflect the time-varying contact rate due to the pupil’s response and countermeasures induced during the outbreak. Although these factors that reduce infection transmission may take into effect at different times, we simply assume that there is a turning point *t*_c_ so that

$$\omega (t)=\{\begin{array}{c} \begin{array}{cc} 1 & t<t_{c} \end{array}\\ \begin{array}{cc} \omega & t\geq t_{c} \end{array} \end{array}$$ (9)

The basic reproductive number before interventions at day *t*_c_ is,

$$R_{0}=\beta (1/\gamma_{1}+1/\gamma_{2})$$ (10A)

and after the collective interventions it becomes

$$R_{c}=\beta \omega (1/\gamma_{1}+1/\gamma_{2})$$ (10B)

We assume the initial conditions as follows: on 10 Mar 2011 there were *I*_0_ infectious but asymptomatic infections (i.e., occult infections) and one symptomatic case (i.e., boy A). For simplicity, we fix the life history intervals of infection (i.e., latent period (1/σ), occult infectious period (1/*γ*_1_) and symptomatic infectious period (1/*γ*_2_)) [16] so that the corresponding serial interval is comparable to the estimate from statistical methods. Priors for other model parameters are assumed to be uniformly distributed with ranges wide enough to include the possible values (Table 3). The variation in the daily number of symptomatic cases is assumed to follow the negative binomial distribution with dispersion parameter *η*. We further assume that the proportion of asymptomatic infections among pupils that were not ill at the end of outbreak follow Poisson distribution. Thus the total likelihood function for estimating the model parameters (*β*, *ρ*, *I*_0_, ω, *t*_c_,*η*) is composed of two parts: negative binomial likelihood that describes the variation on the observation of symptomatic cases {$\bar{D}(t),t=1,\ldots ,90$} from 10 March to 8 June 2011 and Poisson likelihood that describes the variation in positivity of IgG antibodies tests among children that were not ill during the outbreak. It is given by

$$L(\bar{D}|\beta ,\rho ,I_{0},\omega ,t_{c},\eta )=\left(\prod_{t=1}^{90}\frac{\Gamma (\bar{D}(t)+r_{t})}{\Gamma (r_{t})\Gamma (\bar{D}(t)+1)}{\left(\frac{1}{\eta }\right)}^{r_{t}}{\left(1-\frac{1}{\eta }\right)}^{\bar{D}(t)}\right)\left(\frac{\lambda^{\sum_{t=1}^{90}A(t)}\exp(-\lambda )}{\left(\sum_{t=1}^{90}A(t)\right)!}\right)$$ (11)

where

$$r_{t}=D(t)/(\eta -1)$$ (12A)

$$\lambda ={\mathrm{IgG}}^{+}\times (N-\sum_{t=1}^{90}D(t))$$ (12B)

**Table 3:** Parameters of transmission dynamics model.

parameter	definition	Priors	Posteriors median (95% confidence interval)	
			General^a^	Special^b^
*β*_I_	Transmission coefficient	U(0.0029,0.29)	0.128[0.090,0.187]	0.099[0.061,0.123]
*I*_0_	number of occult infections (infectious but not symptomatic) on 10 March 2011	U(0,200)	7.12[0.57,31.8]	0.61[0.03,4.10]
*ω*	Reduction in contact rate	U(0,1)	0·067[0.002,0.431]	0.040[0.002,0.236]
*t*_c_^c^	Turning point in contact rate	U(L/3,L-7)	75.7[67.5,89.4]	74.4[67.0,85.3]
*Ρ*	Proportion of symptomatic infections	U(0,1)	0.085[0.053,0.134]	1.0
*η*	Dispersion parameter	U(1.01,40)	1.71[1.14,3.40]	1.71[1.13, 4.10]
R_0_	Basic reproductive number	–	2.69[1.90,3.92]	2.07[1. 29,2.60]
R_c_	Reproductive number after effective countermeasures	–	0.186[0.006,0.969]	0.079[0.004,0.441]

We assume that the life history durations of infection [16] are latent period (1*/σ*) = 7 days, occult infectious period (1*/γ*_1_) = 14 days and symptomatic infectious period (1*/γ*_2_) = 7 days

^a^ Here we consider the generation situation where infection can be asymptomatic and symptomatic and the proportion of symptomatic infection will be inferred from the model.

^b^ Here we assume all infections are symptomatic.

^c^ L = 90 is the length of outbreak from 10 March to 8 June 2011. *t*_c_ gives the number of days from symptom onset of boy A (10 March 2011)

Hence D(*t*) is the model prediction; IgG^+^ (= 53.5%) represents the positivity of IgG antibodies tests after the outbreak and λ represents the expected number of asymptomatic infection among pupils. *N* (= 698) is the number of pupils at the elementary school [3]. Within the Bayesian framework, Monte-Carlo Markov chain (MCMC) samplings are used to obtain the posterior distribution of model parameters (Table 3).

To test how the results of the analysis depend on the life history stages (latent, occult infection and symptomatic infection) assumed here, we carried out some sensitivity analysis (Table 4). In general, the serial interval can range from the latent period to the sum of latent period and whole period of occult infection and symptomatic infection. Six different combinations of life history stages are considered conditional on a same average serial interval.

**Table 4:** Sensitivity analysis of parameters of transmission dynamics model.

parameter	(7, 14, 7)^a^	(10,13,3)^a^	(10, 11, 7)^a^	(7,11,13)^a^	(7,20,7)^a^	(12,14,7)^a^
*β*_I_	.128 [.090,.187]	.169[.118,.248]	.156[.108,.232]	.122[.087,.171]	.107[.070,.162]	.143[.092,.235]
*I*_0_	7.12 [0.57,31.8]	7.22[0.83,32.5]	6.28[0.38,28.4]	5.81[.54,25.1]	11.9[1.37,51.6]	10.0[0.68,45.6]
*ω*	.067[.002,.431]	.063[.003,.439]	.056[.002,.37]	.052[.002,.327]	.070[.002,.463]	.060[.002,.420]
*t*_c_^b^	75.7 [67.5,89.4]	74.4[67.0,88.3]	75.1[67.2,87.1]	77.0[68.9,87.8]	74.5[67.0,90.8]	73.1[66.9,87.8]
*ρ*	.085 [.053,.134]	.085[.053,.137]	.085[.053,.132]	.085[.055,.133]	.085[.053,.137]	.085[.053,.135]
*η*	1.71 [1.14,3.40]	1.72[1.14,3.46]	1.69[1.12,3.21]	1.66[1.11,3.26]	1.79[1.18,3.58]	1.78[1.15,3.60]
R_0_	2.69 [1.90,3.92]	2.71[1.89,3.97]	2.81[1.95,4.18]	2.93[2.09,4.11]	2.90[1.89,4.37]	3.01[1.95,4.93]
R_c_	.186 [.006,.969]	.176[.007,.946]	.162[.005,.887]	.154[.007,.804]	0.202[.01,1.08]	.188[.01,1.00]

^a^ Life history durations of infection (1*/σ*, 1*/γ*_1_, 1*/γ*_2_) which all assume an average serial interval that is compatible with the mean of the direct estimate (23.9 days) from 15 infector-infectee pairs

^b^ *t*_c_ gives the number of days from symptom onset of boy A (10 March 2011)

## Results

The contact information listed in Table 2 shows that there are 15 cases each of those has a unique likely infector. From these 15 infector-infectee pairs, the serial interval was estimate to have a mean of 23.9 days and standard deviation of 20.9 days and 95% of serial intervals were predicted to lie with the range 1.6 to 79.1 days (this assumes serial intervals come from a Gamma distribution). The same distribution was assumed for the generation time. The maximum likelihood estimate (MLE) from the reconstruction of transmission trees has a mean of 14.4 days and standard deviation of 13.9 days with 95% confidence interval: [0.47, 51.3]. This is shorter than the above direct estimate. The shortness of MLE of serial interval is the consequence of many short pairs of infector-infectee reconstructed: One sample of the reconstructed transmission trees for the Hepatitis A outbreak is shown in the left panel of Fig 1. Within this sample tree, the reconstructed infector-infectee pairs have a mean of 7.7 days, which substantially reduces the overall pooled mean. In view of the large amount of infections being asymptomatic (see the transmission dynamics model below), the shortness of the serial interval of reconstructed infector-infectee pairs must have been due to the ignorance of contribution of asymptomatic infections. Hence we regard the direct estimate as the reasonable serial interval distribution and use it in other analyses.

In order to estimate *R*_0_ in the transmission tree method [9], we sample 10,000 transmission trees from the maximum likelihood estimate of the contact probability *p*_*ij*_. The evolution of effective reproductive number *R*_t_ along the course of outbreak is show in Fig 1 (right panel). In view of the turning point of 26 May suggested from renewal equation (see below), we approximated the average reproductive number during the period from 10 March to 26 May 2011 as *R*_0_ and it is 2.00[1.80,2.20]. If the transmission trees are sampled from the contact probability *p*_*ij*_ generated from Eq (1) using direct estimate of serial interval distribution, we obtained the fairly similar estimate: 2.10[1.9, 2.30] (see Table 1). The underlying reason for the similar results is that *R*_0_ was calculated as the average reproductive number before 26 May 2011 while before this date most infector-infectee pairs were fixed by data rather than being reconstructed by model (Fig 1). With the fully reconstructed transmission tree, we know each case’s position in the pedigree during the outbreak and thus we can easily calculate generation-based reproductive number *R*_g_. This is defined as the average number of infections in the next generation that were generated by the cases in current generation. From the sample tree listed in Fig 1, we found that *R*_g_ reduced not quickly: from initially *R*_g_ = 3 to *R*_g_ = 2.33 with a standard deviation (SD) of 0.94 on the 2^nd^ generation, and *R*_g_ = 1.29 with SD = 1.46 on the 3^rd^ generation. Only on the 4^th^ generation its mean became below the threshold level: *R*_g_ = 0.55 with SD = 0.68. The slow reduction in *R*_g_ over time indicates that there was little early intervention to control the spread of infection.

### Renewal equation method

**Fig 2:**
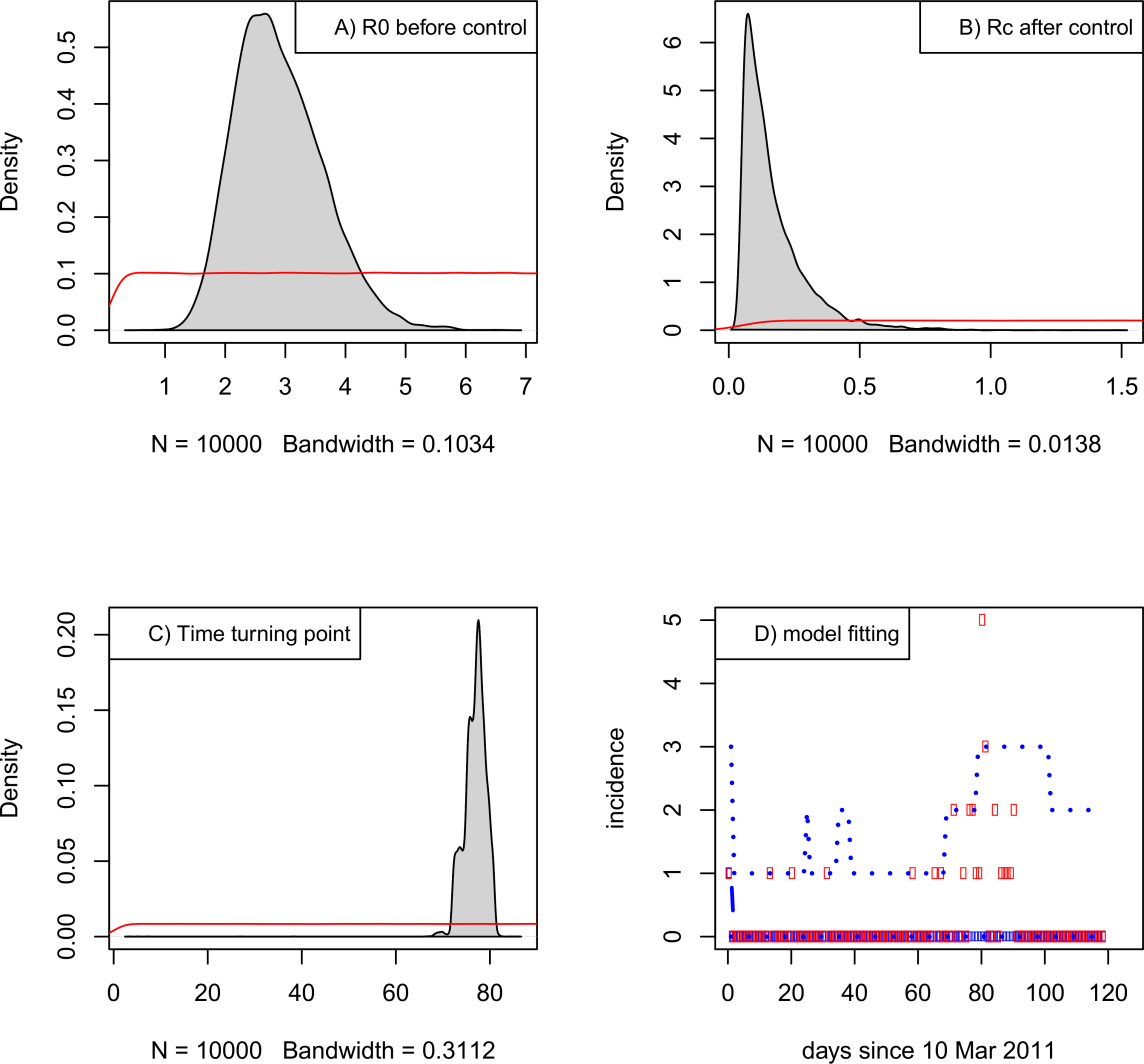
Estimation of parameters of renewal equation model under the assumption of serial interval distribution of mean = 23.9 days and standard deviation = 20.9 days. The Bayesian inference was based on the priors: U(0.1,10), U(0.05,5) and U(1,90) for the three parameters *R*_0_, *R*_c_ and *t*_c_, respectively. Panels A) and B) show the posterior distributions of *R*_0_ and *R*_c_ with red lines for priors. Panel C) shows the posterior distribution of turning point. Panel D) shows model fitting with data (red points): Thick blue dashed lines which mostly overlap with red points denotes median and the thin dashed line represents the upper level of 95% confidence intervals.

Using renewal Eq (4), the MCMC simulations suggest that under the serial interval distribution of mean = 23.9 days and SD = 20.9 days, the countermeasure is collectively started from day 77 with 95% confidence interval [72,81] from 10 Mar 2011 (i.e., 26 May 2011 (22,30 May 2011)), which is just before the peak of the daily incidence (see Fig 2 of [3]). The reproductive number before this turning point is *R*_0_ = 2.82 [1.70, 4.50]; after this point, it reduces to *R*_c_ = 0.17 [0.05, 0.50], indicating transmission is well under control (Fig 2).

### General growth model

**Fig 3:**
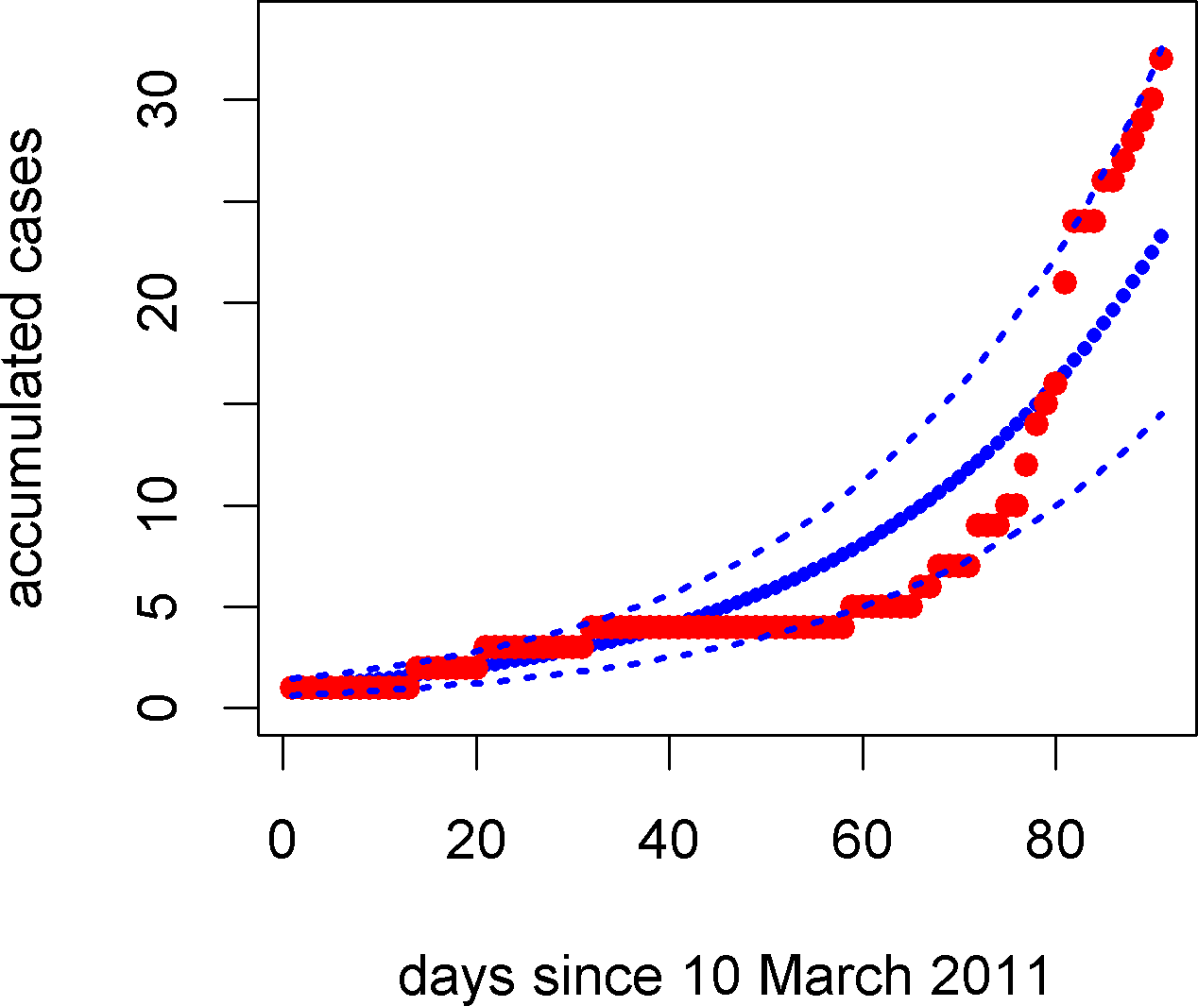
General growth model fitting to data. Red pots represent the observed number of accumulative cases along the course of the outbreak. Thick blue dots are the median predictions and thin dotted lines represent the 95% confidence intervals. The number of initial accumulative case *C*_0_ has median 1.03 and 95% confidence interval [1.00,1.15].

The Bayesian estimates of parameters of general growth model were based on the priors: U(0.002,0.3), U(0,1) and U(0,10) for growth rate, deceleration exponent and the number of initial accumulative cases, respectively. The model fitting is shown in Fig 3. The deceleration exponent *p* is estimated to have a median 0.992 and 95% confidence interval: [0.954,1.0] and it therefore is very close to the unit. This indicates that the exponential growth model is a good approximate to the increase of accumulative incidence in HAV cases. The growth rate has a median 0.0358 and 95% confidence interval ranging from 0.0339 to 0.0379. Assume the serial interval is equal to the direct estimate from observed infector-infectee pairs, 23.9 days, *R*_0_ from Eq (7) is estimated to have a mean 2.35 and 95% confidence interval [2.24,2.48].

### Transmission dynamics model

In view of the serial interval of 24 days, we assume the following values for the progression rates: latent period (1/σ) = 7 days, occult infectious period (1/γ_1_) = 14, symptomatic infectious period (1/γ_2_) = 7days [16]. The analysis results are listed in Table 3. It shows that the turning point occurs at day 76 since 10 March 2011 (with 95% confidence interval ranging from day 67 to day 89). The effect of countermeasures on contact rate is *ω* = 0.067 [0.002,0.431]. The basic reproductive number before effective countermeasures is *R*_0_ = 2.69 [1.90,3.92] and under countermeasures it reduces to *R*_c_ = 0.186 [0.006,0.969]. The initial number of occult infections on 10 March 2011 is about 7 with 95% confidence interval ranging from 1 to 32. The proportion of symptomatic cases among infections is *ρ* = 0.085 [0.053, 0.134]. This implies that at the end of the outbreak, there are about 344 asymptomatically infected pupils along with 32 symptomatic cases.

In the above statistical estimation of *R*_0_, we implicitly assume that all infections are symptomatic. To see whether the ignorance of asymptomatic infections affects the estimation of *R*_0_, we consider in transmission dynamics model a special situation where all infections are assumed to be symptomatic (Table 3). Although the estimate of *R*_0_ decreases under the presumed situation, its mean surely stays within the 95% confidence interval of the true epidemics. To see how the results depend on the assumption of life history stage durations, three other different combinations of three durations were examined (Table 4). The estimates of *R*_0_ are not very sensitivity to the variation in three durations given the same serial interval. It is worth mentioning that the dispersion of the negative binomial likelihood in Eq (11) is very close to the unit for all the above different situations. This justifies Poisson likelihood used in Renewal equation method.

## Discussion

The outbreak of Hepatitis A at an elementary school in Anhui Province, China was evidenced as due to human-to-human transmission. Based on the contact network information provided in [3], serial interval is directly estimated to have a mean of 23.9 days and a standard deviation of 20.9 days. The mean estimates of the transmissibility of hepatitis A virus from three different statistical methods before effective control range from 2.1 to 2.8, with their 95% confidence interval ranging from 1.8 to 4.5. Transmission dynamics model, which includes both asymptomatic and symptomatic infections, generates an estimate of 2.7. When assuming all infections are symptomatic in transmission dynamics model, the estimate reduces to 2.1. These results are well in agreement with statistical estimates which are based on symptomatic case data alone. By further incorporating the results of IgG antibodies test [3], our transmission dynamics model suggests that about 91% of the HAV infections in the school children are asymptomatic. This result suggests that at the end of the outbreak there are total of about 344 asymptomatic infections along with 32 symptomatic cases. This estimate of asymptomatic proportion is consistent with the general estimate [17,18]. The common knowledge is that hepatitis A in children is mostly an asymptomatic disease while adolescents and adults usually show symptoms of clinical hepatitis. For example, one estimation [19] indicates that 80 to 95% of children less than 5 years old have asymptomatic infections, compared to 10 to 25% of adults. A study [20] in Taiwan shows that among children under the age of 10 years, only 10.6% (10/94) of the IgM-anti-HAV positive cases had clinical symptoms.

Our estimates of *R*_0_ are in agreement with that obtained by [21, 22]. Gay et al [21] used serological data collected in England in 1986/7 to estimate average annual incidence in 5–14 year olds and assuming hepatitis A in England was established at endemic equilibrium [6] they estimated *R*_0_ to be 1.6–2.2. Regan et al [21] used a transmission model to investigate the 1991/1992 Sydney outbreak of hepatitis A and estimated *R*_0_ to be 1.7–3.3. Nevertheless, our estimate is higher but still comparable with the estimate of *R*_0_: 1.1–1.6 for hepatitis A in the United States by Van Effelterre et al [23] who used a SEIR compartmental model.

It is worth mentioned that our estimate of serial interval (SI) appears to be short. It is known that people infected with hepatitis A virus experience an incubation period of 28 days ranging 15–50 days to become ill [16,24]. When symptoms occur, they typically last eight weeks [25]. Assuming random mixing and that the latent period is 7 days [16], we have the estimates of serial interval of HAV as SI = 7 + (8–43)/2+56/2 = 39–56 days, taking the middle 48 days as the average. Our direct estimate of serial interval from 15 infector-infectee pairs has a mean of 24 days, which is much shorter than the above guess from experts’ views. The possible explanations for this difference may lie in the fact that the infected persons are all school children at a same elementary school and close and frequent contacts make the transmission quickly. Nevertheless, it should be noted that our estimate has a long tail with 95% confidence interval ranging from about 2 to 79 days, which overlaps with the experts’ guess.

To reflect the reduced growth during outbreaks due to factors such as behavioural change, public health interventions, increased immunity in the population, or any other dynamic change, modifications in simple phenomenological models such as renewal equation model (e.g., [12,26,27]) have been proposed. In this study we introduce an objective time turning point *t*_c_ to indicate the effective and collective start of a variety of countermeasures during the outbreak of hepatitis A in 2011 in Anhui province, China. We hence approximate the average reproductive number before this point as *R*_0_. In general, for any outbreak of infectious disease that originated from one index case and was self-limited or stopped under control, its overall reproductive number throughout the entire outbreak course has a mean equal to (*n*-1)/*n* for an outbreak of size *n*, and its variance can vary from (*n*-1)/*n*^2^ to (*n*-1)^3^/*n*^2^, depending on the structure of the transmission tree (c.f. [28,29]). For the 2011 outbreak in Anhui, the overall mean of reproductive number through the whole outbreak is 0.969, with a standard deviation of 0.106. It is obvious that the overall mean of reproductive number cannot tell us anything about the transmissibility of the infectious agent because it reflects the mixture of the pure transmissibility and countermeasures involved during the outbreak. What we are keen to estimate and is relevant to response plan for policymakers is the transmissibility under somewhat natural conditions (e.g., without changes in humans’ behavior and active control strategies, with all people being susceptible). This simple method of considering growth reduction during the outbreaks can also be applied to the analyses for other outbreaks of other infectious agents [29].

It is well-known that many infections with pathogens such as hepatitis A virus, influenza virus, *Mycoplasma pneumoniae*, and *Mycobacterium tuberculosis* are asymptomatic [4]. For an outbreak caused by such pathogen, the only available data are about symptomatic infections or so at least at the early stage of the outbreak. Theoretically we can argue that if the proportion of symptomatic infections remains unchanged over the outbreak and there is no difference in transmissibility between symptomatic and asymptomatic infections, the estimate of *R*_0_ based only on symptomatic cases should give similar results [8]. In this study we test this for a hepatitis A outbreak using a transmission dynamics model. For this, we investigated two model variants: one includes both types of infection and the other considers only symptomatic infections. We found that the estimates of *R*_0_ from the two model variants, albeit being different, are reasonably in agreement with each other. This is understandable because asymptomatic and symptomatic cases are assumed to be indistinguishable in their infectivity and their life history (i.e. recovery and transition rate to the symptomatic and asymptomatic class), in addition to the assumption of constant proportion of asymptomatic cases over the outbreak. Testing these assumptions is an important research area for future clinical studies. The difference in the estimates of *R*_0_ from the two model variants is also understandable because of the stochasticity in the transmission processes and the limited number of generations during the outbreak.

In conclusion, based on the time series of clinical cases according to symptom onset dates and social networks, we obtain the serial interval and basic reproductive number of hepatitis A virus. These are roughly comparable with previous estimates. We also propose a single turning point during the course of outbreak to identify the objective and collective start of many potential countermeasures and use it to discriminate the transmissibility before effective control and the impact of control strategies. Although Hepatitis A virus can cause both asymptomatic and symptomatic infections, the analysis based on symptomatic infection data can give reliable estimation of transmissibility of Hepatitis A virus.
